# Validation of the Amharic version of Internet Addiction Test-20: a cross-sectional study

**DOI:** 10.3389/fpsyt.2023.1243035

**Published:** 2024-01-09

**Authors:** Nekatbeb Feleke, Awoke Mihretu, Kassahun Habtamu, Beakal Amare, Solomon Teferra

**Affiliations:** ^1^Department of Psychiatry, School of Medicine, College of Medicine and Health Sciences, Bahir Dar University, Bahir Dar, Ethiopia; ^2^Department of Psychiatry, School of Medicine, College of Health Sciences, Addis Ababa University, Addis Ababa, Ethiopia; ^3^School of Psychology, College of Education and Behavioral Studies, Addis Ababa University, Addis Ababa, Ethiopia

**Keywords:** internet addiction, psychometric properties, excessive internet use, pathological internet use, validation, Ethiopia

## Abstract

**Background:**

Internet Addiction is defined as excessive internet use or poorly controlled preoccupations, impulses, or behaviors related to computer use and internet access that cause impairment or suffering. It had devastating effect on people lives, families, productivity, academic performance and rarely engaging in criminal acts like alcohol use, drug addiction, or compulsive gambling. This study aimed to investigate the psychometric properties of the Amharic version of Internet Addiction Test-20 among Addis Ababa University, College of Health Sciences medical students, Addis Ababa, Ethiopia.

**Methods:**

A cross sectional study was carried out among 410 medical students using a convenience sampling method after stratifying them based on their year level. SPSS Version 23 was used to do Pearson’s correlation coefficient to determine the convergent validity of Amharic version of IAT. We computed correlation coefficient between the aggregate scores of IAT-20 and the scores for depressive symptoms, problematic substance use, and other characteristics of participants which was assessed using Patient health questionnaire-9 (PHQ-9), Alcohol, Smoking and Substance Involvement Screening Test (ASSIST) and questionnaire developed to assess demographic and internet use related characteristics, respectively. AMOS 23 software was used to conduct confirmatory factor analysis (CFA) to evaluate the construct validity of Amharic version of IAT. Test–retest reliability was also determined with 2 weeks interval (*n* = 51).

**Results:**

The data confirmed a two-factor structure. Normed Fit Index (NFI) = 0.89, Tucker Lewis Index (TLI) = 0.91 and Comparative Fit Index (CFI) = 0.92, Root Mean Square Error Approximation (RMSEA) = 0.07, and Standardized Root Mean Residual (SRMR) =0.05 indicated a good fit model structure. There was moderate positive correlation between the aggregate scores of IAT-20 and PHQ-9 scores (*r* = 0.55, *p* < 0.00), but weak positive correlation between IAT-20 and ASSIST scores (*r* = 0.14, *p* < 0.00). IAT-20 was also found to have good internal consistency (Cronbach’s alpha = 0.89 for each factor). The test–retest reliability was also good for all items (Intra Class Correlation Coefficient (ICC) > 0.30) except item 16.

**Conclusion:**

We found that the IAT-20 is psychometrically sound and a simple screening test for Internet Addiction. However, it is important to acknowledge that further studies are necessary to replicate these findings on diverse population.

## Introduction

The global internet penetration rate is estimated to be 66.2% (1); with Africa and Ethiopia having rates of 43.1% and 17.9%, respectively (2). Internet Addiction (IA) is defined as excessive or poorly controlled preoccupations, impulses, or behaviors related to computer use and internet access that cause impairment or suffering (3); It is a prevalent problem issue globally, with an overall estimate of 6.0% (4). However, IA is not currently recognized as a disorder in the Diagnostic and Statistical Manual of Mental Disorders, Fifth Edition, Text Revision (DSM-5-TR) or the International Classification of Diseases-11 (ICD-11) (5, 6).

While the internet offers enormous benefits, including facilitating knowledge acquisition, enhancing health, and performing procedures remotely, it also poses problems when its use is problematic, has been linked to various types of psychological distress, such as insomnia, anxiety, depression, conduct problems, hyperactivity, poor quality of life, and low self-esteem (7, 8). It has also been associated with psychiatric symptoms such as somatization, obsession and compulsion, interpersonal sensitivity, depression, anxiety, hostility, phobic anxiety, paranoid ideation, and psychoticism (7). IA is a major public health concern among African university students, with approximately one-third exhibiting features of addiction (9). Being male, residing in urban areas, and spending four or more hours online were the commonly reported associated factors of internet addiction (9).

Currently, gaming disorder, both online and offline, is recognized in the ICD-11 as a class of disorders due to addictive behaviors, and internet gaming disorder is listed under conditions for further study in the DSM-TR (5, 6). IA had devastating effect on their lives, families, productivity, academic performance and rarely engaging in criminal acts like alcohol user, drug addiction, or compulsive gambling (10, 11).

Internet addiction (IA), problematic internet use, and excessive internet use are similar constructs that have been investigated in recent years. The most widely used tool to screen for problematic internet use is the Internet Addiction Test-20 (IAT-20) (12). IAT is a 20-item Likert scale that assesses the presence and severity of adult internet dependency (10).

In Ethiopia, although the number of internet users has significantly increased (2), there is insufficient evidence of internet addiction using a valid screening tool. Internet addiction was reported to be 19.4% among Dilla University students, 85% among Wollo University students, and 28.2% among Addis Ababa University students, the first two used English version of IAT-20 and the third one used Amharic version of IAT-20 using expert opinion after translation (13–15). Although these studies focus on university students, the findings vary greatly. The reason for these variations in the prevalence of internet addiction among university students in Ethiopia may be due to the use of IAT-20 test which have not been validated in the Ethiopian context. Although IAT-20 has been frequently used, its cultural appropriateness and psychometric properties have not been established in the Ethiopian context, specifically among university students. Validating IAT-20 in Ethiopia will facilitate future epidemiological studies and inform interventions. Therefore, the current study aims to validate the Internet Addiction Test (IAT-20) among university students in Ethiopia.

## Methods

### Study design

We conducted a cross-sectional study to determine the psychometric properties of the Amharic Version of the Internet Addiction Test (Amharic version of IAT) among university students in Addis Ababa, Ethiopia.

### Study area

The study took place at Addis Ababa University (AAU), the oldest and largest public university in Ethiopia. The College of Health Sciences (CHS) is one of the ten colleges at AAU, founded in 2009/10. CHS consists of one teaching hospital and four schools: the School of Medicine (SoM), the School of Pharmacy (SoP), the School of Public Health (SPH), and the School of Allied Health Sciences (SAHS). All of the CHS schools, except the School of Public Health, offer professional degrees at both the undergraduate and graduate levels. Currently, CHS has more than 5,000 students and over 600 full-time academic staff. We targeted undergraduate medical students for our study as the SoM offers undergraduate degree programs in Medicine, Dental Medicine, Anesthesia, and Radiology and has a large population size for our study.

### Study population sample size and sampling procedure

We targeted undergraduate medical students at AAU, CHS for this validation study. For validation studies, a sample size of 300–500 is recommended to conduct confirmatory analysis (16) and based on the rule of thumb that 5–20 participants per item are necessary for validation studies involving confirmatory factor analysis (17–19). Therefore, using the highest recommended sample size per variable or item, we took a sample size of 420 participants, including a 5% non-response rate. Participants were stratified by their academic year level and they were selected using convenience sampling technique among students attending the class during the time of data collection. For the test–retest, we included a sub-sample of 60 participants and their anonymity was ensured by coding their names’ first letter and their phone numbers’ last five digits. We administered the test–retest after 14 days of the first administration. For the test–retest, 60 participants were involved, and their anonymity was ensured by coding their names’ first letter and their phone numbers’ last five digits. We administered the test–retest after 14 days of the first administration (20).

## Measures

### Socio-demographic and internet use related characteristics

We developed and used a structured questionnaire to collect data on selected socio-demographic and internet use related characteristics, including sex, age, origin of residence, year of study, self-reported academic performance, means of internet access, purpose of internet use, amount of time spent online, and type of internet and the type of internet devices participants use.

### Internet Addiction Test (IAT-20)

The IAT-20, a Likert-type scale, is designed to evaluate specific symptoms of problematic internet use, which are similar to other recognized compulsions such as gambling, eating, and sex. The test determines the level of an individual’s internet use and categorizes internet addictive behavior as normal, mild, moderate, or severe, depending on the IAT-total score: 31–49, 49–80, and 80 and above, respectively (10). However, different studies have reported varying cut-off scores to indicate internet addiction or problematic internet use.

The IAT is a globally recognized and validated instrument that can be used in the general population, as well as in both outpatient and inpatient settings, and can be customized to meet individual needs (21–23). The IAT’s items were identified from previous research and clinical studies that investigated many aspects of online activity and identified qualities that distinguish “normal” from compulsive online users (10). The IAT is intended for experienced internet users who make extensive use of the technology. Each statement is weighted along a Likert-scale ranging from 0 to 5, with 0 being the least extreme conduct and 5 representing the most extreme behavior (10).

On different studies researcher used different cut of scores points to indicate internet addiction or problematic internet use, for this study we used the cut of point proposed by the developer. Problematic internet use or Internet addiction will be classified normal, mild; moderate and severe depending IAT-total score 0–30; 31–49; 49–80; and 80 and above, respectively (24).

The IAT total score is the sum of the ratings given by the examinee for the 20 item responses, a 5-point scale ranging from minimum score of 0 to a maximum score is 100 points. Research addressing the sensitivity and validity of these score ranges is published in several journals (10). IAT was assessed for psychometric properties in different languages of Europe countries; French, Italy; Greece; Germany, Portuguese, Spanish and found to be a valid instrument to assess internet addiction (22, 25–29). It was translated and validated to different languages in countries like Bangladeshi; Lebanon; Malaysia; Pakistan and Korean (30–34). In Africa setting IAT-20 was validated in Nigeria (35). In all the above settings IAT-20 was found to be a valid and reliable instrument to assess IA.

### Internet Addiction Test adaptation

The translation of the original IAT-20 from English into Amharic was conducted using recommended established procedures (36). First, the IAT-20 was independently translated from English to Amharic by two translators. Then, the translated English language version was independently back-translated by other translators into English. Finally, the translated versions were evaluated and reached consensus among translators and mental health and psychometric experts.

The language translators’ team agreed to use the term “Beyne Mereb” as the Amharic language equivalent of the English word “internet.” We also rephrased Item 3, which stated about internet use and intimacy with friends, and Items 6 and 8, which were about productivity or school performance. We rephrased Item 8 to assess general productivity, including extracurricular tasks, and Item 6 specifically to academic performance. Items 3 and 19 were also rephrased. Item 3 now reads “how often do you prefer the internet over your relationships,” while Item 19 assesses students’ preference to use their free time on the internet more than with a close person. Item 7, which initially stated “how often do you check your email,” was changed to “how often did you check the internet (e.g., social media and others) before anything else,” reflecting the increased means of being online. We further explored the readability and formatting issues of the IAT-20 by administering the scale to a sample of 12 students. They were informed to report questions they had difficulty understanding and other typographical errors, but they did not report any difficulty with the AV-IAT-20 and only suggested the inclusion of TikTok in the choices of applications. The Amharic version of the IAT-20 test was then administered to a larger sample.

### Depression

We used Patient Health Questionnaire (PHQ-9) to assess convergent validity because depression and internet addiction in previous studies (37–39). The nine items (PHQ-9) was developed to screen depressive symptoms in the general population (40). Then PHQ-9 has been validated in Ethiopia, both in rural and urban settings and acceptable psychometric properties. The PHQ-9 is found to have one factor structure, Cronbach’s alpha = 0.81, test re-test reliability (ICC = 0.92), sensitivity of 86% and specificity of 67%, in the Ethiopian context (41, 42).

### Substance use

We used the Alcohol, Smoking and Substance Involvement Screening Test (ASSIST) because it substance use was associated with internet addiction (43–45). We used ASSIST for risky use of psycho active substances (Tobacco, Khat, Alcohol and Others). The ASSIST was developed by the World Health Organization (WHO) and an international team of substance use researchers as a simple method of screening for hazardous, harmful, and dependent use of alcohol, tobacco and other psycho active substances (46). The Amharic version of the ASSIST was administered to assess problematic alcohol, tobacco, and khat use. The tool has been frequently used in Ethiopia, and the current study found an internal consistency (Cronbach’s alpha) of 0.93 but it has not been validated in Ethiopian setting.

## Data quality assurance

We provided detailed orientation to the data collectors. The principal investigator supervised the entire data collection process. The primary author also closely conducted data entry and analysis with one of the co-authors.

## Data analysis

To assess the internal consistency and reliability of the Amharic version of IAT, we used Cronbach’s alpha. Values between 0.70 and 0.80 are considered fair, while values between 0.80 and 0.90 indicate good internal consistency. We used the Intraclass Correlation Coefficient (ICC) to determine test–retest reliability. ICC estimates less than 0.0 indicate poor fit, while estimates between 0.0 and 0.20, between 0.21 and 0.40, 0.41–0.60, 0.61–0.80, and 0.81 are indicative of poor, slight, fair, moderate, good, and excellent reliability, respectively (47).

We utilized the Pearson correlation coefficient to assess the convergent validity of the IAT-20 using its correlation with PHQ-9, ASSIST, duration of online hours per day and staying late online to use internet as those are frequently associated with problematic internet use (37, 48–50); for continuous and point biserial correlation for Tiktok and peer pressure. Prior to conducting the convergent validity analysis, we rigorously examined essential assumptions, including linearity, normality, and the nature of the variables.

We conducted confirmatory factor analysis (CFA) to examine the factor structure IAT-20. CFA was carried out to test AV-IAT’s factor structures that were proposed in previous studies: one factor (22, 51), two factors (25, 27, 29, 52), 3 factors (26, 53), 4 factors (31, 54) and 6 factors (10). We used the following indices to assess the goodness of fit: the *χ*^2^ test, acceptable if *χ*^2^/df is less than 3.0; Comparative Fit Index (CFI), acceptable if its value ≥ 0.95; Tucker-Lewis Index (TLI), acceptable if its value exceeds 0.95; Root Mean Square Error of Approximation (RMSEA), acceptable if the value is close to 0.06 (55, 56) and Standard Root Mean Squared Residual (SRMR), acceptable if it is <0.08 (56).

## Ethical considerations

The study protocol was approved by Research Ethics Committee of the Department of Psychiatry, College of Health Sciences, Addis Ababa University (ref. MF/PSY/1179/14). Information sheet was prepared and presented to each participant to allow for a free and informed decision to participate in the study. Informed consent was taken in written form. We ensured privacy and confidentiality during data collection, handling, and reporting. We also adhered to all standard national guidelines for COVID-19 precautions, including wearing a facemask, physical distancing, and taking hand hygiene measures.

## Results

### Socio-demographic and internet use related characteristics of participants

In total, 410 participants (i.e., a 97.6% response rate) were included in this study. We excluded ten incomplete questionnaires from the analysis. Fifty-one of the participants involved in a repeat administration of the AV-IAT-20 to determine test–retest reliability. The mean (±SD) age of the participants was 23 years (±1.98), and nearly half (47.1%, *n* = 193) were female. Approximately 17% (*n* = 71) of the participants came from rural areas. Regarding the year level, 13.2% (*n* = 54) were in their second year of study, 12.2% (*n* = 50) in their third year, 20.7% (*n* = 80) in their fourth year, 23.9% (*n* = 98) in their fifth year, and 30% (*n* = 123) in their final year. Of all the participants, 92% (*n* = 377) used the internet for research and academic purposes, 37.1% (*n* = 152) for playing online games, 87.1% (*n* = 357) for watching online videos, and 82.4% (*n* = 338) for social media. Hence, most of the students used the internet for multiple purposes.

Most of the participants accessed the internet through their mobile phones (94.6%), while only 40% (*n* = 164) used tablets. The mean (±SD) time spent online per day was 4 h (±2.143), with a range of 30 min per day to 14 h per day. In addition, 21.9% (*n* = 94) of participants reported staying up late at night to use the internet. The most common mode of internet access was Wi-Fi (90.2%, *n* = 370), followed by mobile data (32.7%, *n* = 134) and broadband (3.2%, *n* = 13). The most commonly used application for internet use was YouTube (79.5%, *n* = 376), 65.3% (*n* = 267) used three or more applications at a time (Table 1). About 10.7% (*n* = 44) of the participants reported having low social support, while around 21% (*n* = 86) reported peer pressure as a pushing factor for internet use.

**Table 1:** Socio-demographic and internet use related characteristics of the medical students (*N* = 410).

Variable	Category	*n*	%
Gender	Male	217	52.9
	Female	193	47.1
Age	Mean (±SD)	22.9 (±1.982)	
Year of study	2nd (PC-I)	54	13.2
	3rd (PC-II)	50	12.2
	4th (C-I)	85	20.7
	5th (C-II)	98	23.9
	6th (Internship)	123	30
Self-reported academic performance	Low	12	2.9
	Fair	100	24.4
	Good	181	44.1
	Very good	95	23.2
	Excellent	21	5.1
Time spent online per day	Mean (±SD)	4 (±2.14)	
Most commonly reported purpose for internet use	Academic and research purpose	377	92.1
	Watching videos	357	87.1
	Online games	152	37.1
	Forming friendship	163	39.8
	For news online	261	39.8
Years since being online	Mean (±SD)	8.2 (±2.90)	
Most commonly used app	YouTube	326	79.5
	Telegram	317	77.3
	Surfing engines google …	281	68.5
	Facebook	103	25.1
	Tiktok	134	32.7
	Other apps	20	4.9
Social support	Low	44	10.7
	Moderate	250	61
	High	116	28.3
Self-reported peer pressure	Low	322	78.5
	High	86	21
Staying late for internet use	Never	62	15.1
	Rarely	254	62
	Usually	87	21.2
	Always	7	1.7
Means of internet access	Wi-Fi	370	90.2
	Mobile data	134	32.7
	Broad band internet		
	Band	13	3.2
Materials used for internet access	Phone	388	94.6
	Tablet	164	40
	Computers	331	80.7

PC-I, Preclinical year one; P-II, Preclinical year two; C-I, Clinical year one; C-II, Clinical year two.

### Descriptive statistics of the Internet Addiction Test

Descriptive statistics of the Internet Addiction Test (IAT-20) revealed that the item mean for Factor 1 was 1.93, and 1.77 for Factor 2. The highest mean score was 2.78 (Item 1), which indicated a tendency to stay online longer than intended, while the lowest mean score was 1.19 (Item 5), indicating that others did not complain about the amount of time spent online. The highest Item-total correlation was 0.73 for Item 8 (How often does your job performance or productivity suffer because of the internet?), and the lowest was for Item 4 (0.42) (How often do you form new relationships with fellow online users?). Using the cutoff point proposed by Young (10), about 2.4% (*n* = 10) had a severe level of problematic internet use, while 15.6% (*n* = 64) had a moderate level of problematic internet use.

## Reliability and validity (semantic/language, construct/convergent, CFA) of IAT

### Reliability

The internal consistency reliability (Cronbach’s alpha) of AV-IAT-20 was 0.93. Internal consistency reliability was not increased significantly when calculated removing each item. Test–retest reliability of each item using Intra class correlation coefficient (ICC) ranging between 0.79 and 0.3; ICC all of the items were greater than 0.30 except Item16. The highest was Item8 (ICC = 0.79) and the lowest was, Item16 (ICC = 0.12) (Table 2).

**Table 2:** Mean value, standard deviation, item-total correlation, Cronbach’s alpha and test–retest reliability of items.

Items	Item code	Mean	SD	Corrected ITC	α If item deleted	ICC	Cronbach’α
How often do you find that you stay online longer than you intended?	IAT1	2.78	1.17	0.53	0.930	0.63	
How often do you neglect household chores to spend more time online?	IAT2	2.19	1.20	0.69	0.927	0.72	
How often do you form new relationships with fellow online users?	IAT4	1.28	1.03	0.42	0.932	0.58	
How often do others in your life complains to you about the amount of time, you spend online?	IAT5	1.19	1.17	0.65	0.928	0.52	
How often do your grades or school work suffer because of the amount of time you spend online?	IAT6	1.59	1.36	0.70	0.927	0.37	
How often do you check your email before something else that you need to do?	IAT7	2.31	1.42	0.65	0.928	0.77	
How often does your job performance or productivity suffers because of the Internet?	IAT8	1.79	1.30	0.73	0.926	0.79	
How often do you lose sleep due to being online?	IAT14	1.67	1.18	0.65	0.928	0.73	
How often do you find yourself saying “Just a few more minutes” when online?	IAT16	2.60	1.32	0.65	0.928	0.12	
How often do you prefer the excitement of the internet to intimacy with your partner?	IAT3	2.18	1.38	0.60	0.929	0.70	
How often do you become defensive or secretive when anyone asks you what you do online?	IAT9	1.23	1.32	0.57	0.929	0.72	
How often do you block out disturbing thoughts about your life with soothing thoughts of the Internet?	IAT10	1.79	1.43	0.54	0.930	0.61	
How often do you find yourself anticipating when you will go online again?	IAT11	2.03	1.33	0.65	0.929	0.59	
How often do you fear that life without the Internet would be boring, empty, and joyless?	IAT12	1.32	1.46	0.56	0.930	0.69	
How often do you snap, yell, or act annoyed if someone bothers you while you are online?	IAT13	1.40	1.16	0.58	0.929	0.69	
How often do you feel preoccupied with the Internet when off-line, or fantasize about being online?	IAT15	1.31	1.19	0.65	0.928	0.65	
Do you try to cut down the amount of time you spend online and fail?	IAT17	2.07	1.39	0.68	0.927	0.63	
How often do you try to hide how long you have been online?	IAT18	1.33	1.37	0.60	0.929	0.39	
How often do you choose to spend more time online over going out with others?	IAT19	1.84	1.37	0.57	0.929	0.67	
How often do you feel depressed, moody, or nervous when you are off-line, which goes away once you are back online?	IAT20	1.47	1.25	0.66	0.927	0.60	
Total items—20							0.93
F1							0.89
F2							0.89

SD, Standard deviation; ICC, Intra Class Correlation Coefficient; ITC, Item total correlation; α, Alpha; F1, Factor 1 and F2, Factor 2.

### Convergent validity

Convergent validity was assessed using Pearson’s correlation coefficient to see correlation between total score of AV-IAT-20 and PHQ-9, ASSIST, duration of online hours per day, staying late online at night to use internet, self-reported academic performance and social support. We used point biserial correlation conducted for dichotomous variables. There was moderate correlation between scores of AV-IAT-20 and PHQ-9 (*r* = 0.55; *p* < 0.00). There was weak positive correlation between scores of AV-IAT-20 and ASSIST (*r* = 0.14; *p* < 0.00). There was weak positive correlation between duration of online hours per day and AV-IAT-20 scores (*r* = 0.30, *p* < 0.00), staying late online at night to use internet and AV-IAT-20 scores (*r* = 0.42, *p* < 0.001). There was a weak negative correlation between AV-IAT-20 and social support (*r* = −0.18, *p* < 0.00). Among the dichotomous variables, there was weak positive correlation between Tiktok use and AV-IAT-20 score (*r* = 0.18, *p* < 0.00), and between peer pressure and AV-IAT scores (*r* = 0.19, *p* < 0.00) (Table 3).

**Table 3:** Pearson’s correlation between IAT-20 score; PHQ-9 score, ASSISST score.

	IAT score	PHQ-9	ASSISST score	Duration of online hrs/day	Self reported academic performance	Having social support	Staying late at night to use internet
IAT score	1						
PHQ-9	0.55*	1					
ASSISST score	0.14*	0.18	1				
Duration of online hrs per day	0.30*	0.24*	0.17	1			
Self-reported academic report	−0.19*	−0.18*	0.04*	−0.02	1		
Having social support	−0.22*	−0.04	0.01	0.04	0.10*	1	
Staying late at night to use internet	0.42*	0.23*	0.14*	0.29*	−0.04	−0.037	1

*p < 0.05.

### Confirmatory factor analysis

**Figure 1:**
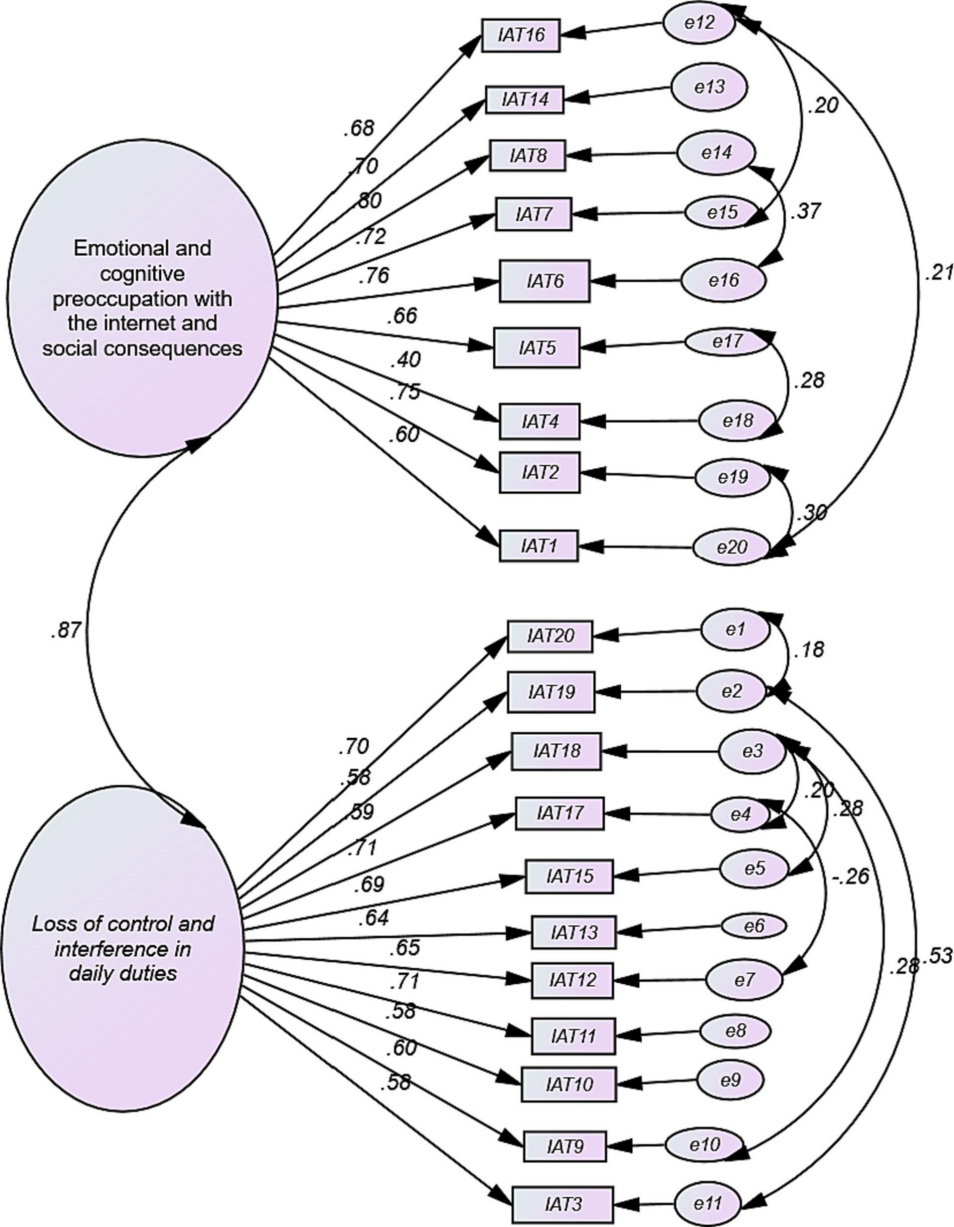
Confirmatory factor analysis diagrammatic path of Amharic version of Internet Addiction Test-20.

Confirmatory factor Analysis (CFA) was performed after the Kaiser-Meyer-Olkin measure of sampling adequacy (0.94) was determined and Bartlett’s test of sphericity was significant (*χ*^2^ = 4,294.8, *df* = 190, *p* < 0.05), which indicate that the items are suitable for CFA analysis. All of the 20 items highly loaded into their respective factors with an item factor standardized loading of 0.58 and above, except item 4 (with item factor standardized loading of 0.40) (Figure 1).

CFA was iteratively conducted on each of the proposed factor structures. The result showed that none of the factor structures fit as indicated by NFI, TLI, CFI, RMSEA and SRMR (Table 4). For this reason, we did modification indices considering to make correlations for all of the proposed factors. After correlating the factors, the CFA of two factor structure resulted *χ*^2^ = 853.79, *df* = 169 and *χ*^2^/df = 5.05. The values of NFI = 0.89, TFI = 0.90 and CFI = 0.92, RMSEA were also found to be less than the recommended parameter (<0.08) and that of SRMR = 0.05 (close to 0.05). Therefore, the construct is considered good fit. Hence, we declare that the CFA findings of the AV-IAT-20 has 2-factor structure. The one factor structure also showed a good fit model, but it needs extensive correlation of errors so that we decided to drop the model (Supplementary Figure S1).

**Table 4:** Comparison for good fitness obtained from the confirmatory factor analysis from different factor structures.

Factors	IAT-version	Item	*χ* ^2^	df	*χ*^2^/df	NFI	TLI	CFI	RMSEA	SRMR	Covariance between latent variable range
1	United States, French, and Arabic	20	1045.543*	170	6.149	0.761	0.766	0.791	0.112	0.0734	
2	United States and German	20	793.502*	169	4.694	0.819	0.832	0.851	0.095	0.0635	0.81
	Spanish	19	806.163	151	5.339	801	809	0.831	0.103	0.0694	0.0694
	Italian	20	853.790*	169	5.052	0.805	0.916	0.836	0.100	0.0678	0.83
	India	17	517.541*	118	4.386	0.858	0.868	0.886	0.085	0.0604	0.91
3	Greek	20	810.301*	167	4.852	0.815	0.825	0.846	0.097	0.0701	0.77–0.92
	Hong Kong	18	661.099*	132	5.01	0.824	0.830	0.853	0.099	0.0630	0.79–1.0
4	Lebanese	20	918.942*	164	5.603	0.790	0.91	0.820	0.106	0.0711	0.82–1.0
	Turkish	20	823.177*	164	5.02	0.812	0.818	0.842	0.099	0.0681	0.72–0.93
	Bangladesh	18	661.018*	129	5.124	0.823	0.824	0.85	0.100	0.0649	0.77–0.98
6	United States	20	843.879*	155	5.444	0.807	0.798	0.835	0.104	0.0722	0.68–1.0

*p < 0.001. RMSEA, Root Mean Error Approximation, Standardized Root Mean residual; NFI, Normative Fit Index; TFI, Tucker Lewis Index; CFI, Comparative Fit index.

## Discussion

The aim of this study was to examine the psychometric properties of the Amharic version of IAT-20. The study found Amharic version of IAT-20 has good convergent validity. We found moderate correlation between IAT scores and depressive symptoms in the expected direction. Confirmatory factor analysis showed that the Amharic version of IAT-20 fits into a 2-factor solution. Amharic version of IAT-20 is also found to have excellent internal consistency and moderate test–retest reliability.

The current study found that the Amharic version of IAT is a valid and potentially useful instrument for undergraduate medical students this is consistent with reports from previous studies (32, 33). This study has shown to have excellent internal consistency reliability; for the total scale (Cronbach’s α = 0 0.93) and for each factor (Cronbach’s α =0 0.89). The finding is consistent with a previous validation study which found excellent internal consistency reliability (Cronbach’s α = 0.93) (22, 25, 57–59). The two-weeks test retest-reliability is found to be moderate for all of the items except item 16 and this is consistent with a previous report (27).

In this study, we compared different proposed factor structures ranging single factor solution to six factor solution. The analysis confirmed a two-factor solution better fitted the data. Prior studies indicated that several IAT models; one-factor model to six-factor model (22, 24, 28, 30, 31, 53, 54, 59–63) which had different Items in the latent variables even with in the same factor structures (22, 25, 29, 58, 64). This suggests that the number of factors of IAT is not well established cross-culturally. It sounds as if there needs to be more inductive work to understand how internet addiction is manifested in different cultures (including in Ethiopia). After conducting CFA, we considered the 2-factor solution is consistent for our data (see Table 4). The first factor is “Emotional and cognitive preoccupations with the Internet and social consequences” and the second factor is “Loss of control and interference with daily duties.” These names also work for the current study findings.

In the CFA we did, all the Items loaded on their respective factors, with factor loading ranging from 0.57 to 0.79; except Item4 which loaded 0.40. Item4 was found to have a lower or fair range of factor loading in other previous studies (25, 27). Item4 showed the lowest item-total correlation and the lowest factor loading, probably because it refers to a common behavior (i.e., forming relationships online) that cannot be necessarily considered a problematic behavioral symptom of internet addiction rather it might be considered as a normal behavior. Networking through the internet and forming relationships online might be considered acceptable (58, 59).

The Amharic version of IAT showed good convergent validity which was in line with previous studies (27). There was moderate positive correlation between IAT scores and PHQ-9 scores which is consistent with the finding of previous studies (37, 65). There was also a small correlation with those who use Tik Tok and small negative correlation between having good social support and IAT-20 scores. There is small negative correlation with cumulative GPA and a positive correlation with self-reported academic performance. This might be because of the procrastination for internet use in students (50). There is also correlation of IAT scores and duration of online hours per day, hours spent online per week and spending more than 4 h. These findings go with previous studies (9).

There was moderate positive correlation between staying late at night to use internet and IAT score, which is consistent with previous study (66); though it contrasts to another previous study (67). Thus, the finding related to staying late for internet use needs careful interpretations.

This is study is not without limitation. The first limitation of this study is conducting CFA before exploratory factor analysis. Second, College students are a key population in which intense Internet use is common. Therefore, generalizing results to other adult populations may not be possible. Nevertheless, this study is still valuable as the first investigation to report the reliability and validity of the Amharic version of IAT. In recent times the importance of IA is getting more attention as evidenced by internet gaming disorder in DSM-TR. As more cross-cultural validations of the Amharic version of IAT accumulate, we would be able to assess internet addiction and able to measure IA consistently using this valid tool.

## Conclusion

The Amharic version of the IAT has demonstrated strong psychometric properties, suggesting good reliability and validity in assessing problematic internet use among Ethiopian university students. Thus, it can be effectively utilized as a screening tool for internet addiction among this population. However, it is important to recognize the need for further evaluation of its use beyond this group. In light of the increasing reliance on the internet, particularly among young people in our society, this study serves as an essential starting point for developing effective means to design epidemiological and intervention studies.

## Data availability statement

The raw data supporting the conclusions of this article will be made available by the authors, without undue reservation.

## Ethics statement

The studies involving humans were approved by Research Ethics Committee of the Department of Psychiatry, College of Health Sciences, Addis Ababa University. The studies were conducted in accordance with the local legislation and institutional requirements. The participants provided their written informed consent to participate in this study.

## Author contributions

NF, AM, ST, and BA conceived, performed and designed the study. NF, AM, ST, BA, and KH contributed on data analysis and manuscript write-up. All authors contributed to the article and approved the submitted version.
