# Supplementary material for: Validation of the Amharic version of Internet Addiction Test-20: a cross-sectional study
Source: Front Psychiatry. 2024 Jan 9;14:1243035. doi: 10.3389/fpsyt.2023.1243035 (PMC10803712; doi:10.3389/fpsyt.2023.1243035)
Supplement: Supplementary file 1 [file Data_Sheet_1.docx]

**
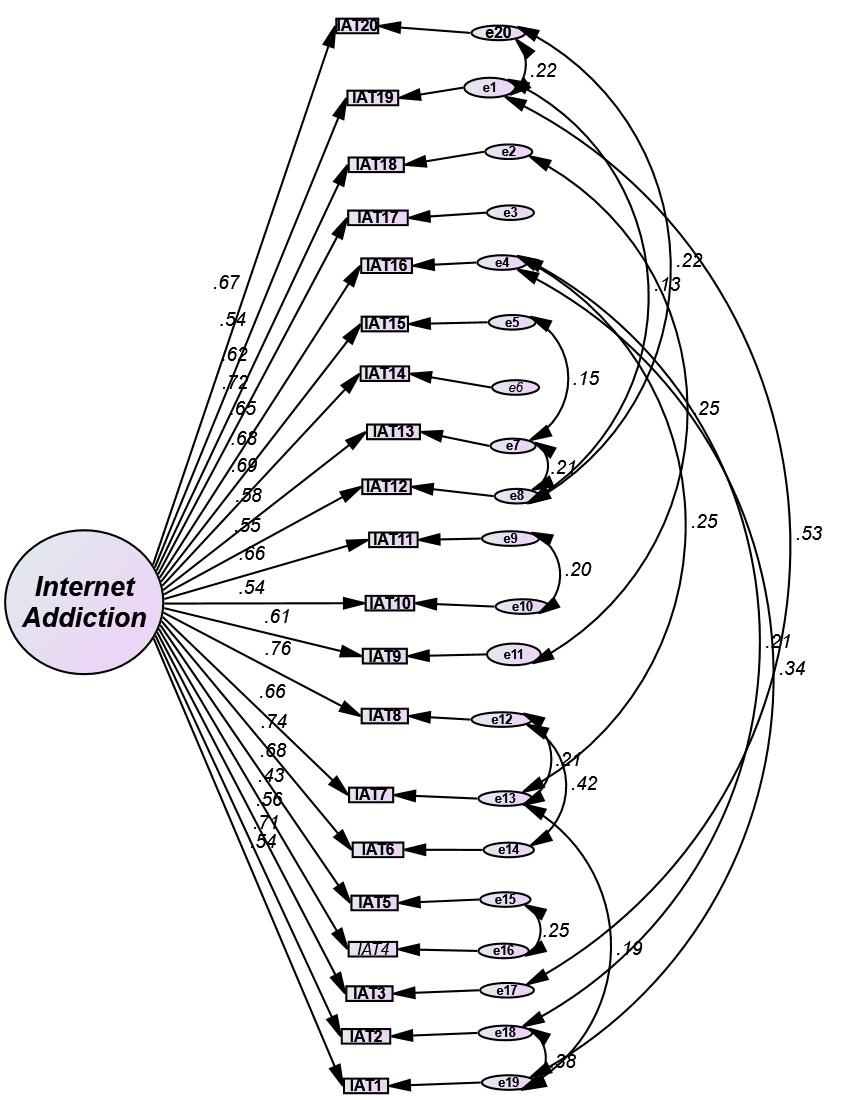
**

Supplementary Figure 1: Confirmatory factor analysis diagram using one factor model of Amharic Version of Internet Addiction Test -20
